# The cross-cultural validity of the Resilience Scale for Adults: a comparison between Norway and Brazil

**DOI:** 10.1186/s40359-015-0076-1

**Published:** 2015-06-18

**Authors:** Odin Hjemdal, Antonio Roazzi, Maria da Graça B. B. Dias, Oddgeir Friborg

**Affiliations:** Department of Psychology, Norwegian University of Science and Technology, Trondheim, Norway; Department of Psychology, Cidade Universitária, Recife, Pernambuco Brazil; Faculty of Health Sciences, Department of Psychology, UiT The Arctic University of Norway, N-9037 Tromsø, Norway

**Keywords:** Resilience, Resilience scale for Adults, Cross-cultural validation, Sense of Coherence, HSCL-25

## Abstract

**Background:**

The resilience construct is of increasing interest in clinical and health psychology. The Resilience Scale for Adults (RSA) is a measure of protective factors. The evidence supporting its construct validity is good, however evidence of cross-cultural validity is modest.

The present study explored the factorial invariance of the RSA across a Brazilian and a Norwegian sample, as well as the construct validity in the Brazilian sample.

**Methods:**

The Brazilian sample (*N =* 222) completed the Hopkins Symptom Check List-25 (HSCL-25), the Sense of Coherence (SOC), and the RSA. The Norwegian sample (*N* = 314) was included in order to examine the factorial invariance.

**Results:**

The results indicated that the latent constructs of the RSA (its primary factors) are the same in the Brazilian sample as in the Norwegian sample. The correlations between the subscales of the RSA were significant. In the Brazilian sample, the correlations with HSCL-25 and SOC were negative and positive, respectively, thus supporting its construct validity.

**Conclusion:**

The results indicate that the original factor structure of the RSA based on Norwegian samples remains stable in a Brazilian sample.

## Background

The World Health Organization estimates that mental disorders affect some 450 million people at any given moment (WHO, 2001). In addition to focusing on risk and vulnerability, identification and measurement of protective factors are important for widening our understanding of mental health (Masten, 2011). A proper assessment of protective factors are however challenging because their importance and relevance may vary across samples (e.g., healthy versus patients), different life circumstances (e.g., exposure to trauma, losses or other negative life events), but also across nations and cultures. There have been a few attempts of generating self-report measures of protective factors based on resilience research. A critical evaluation in 2011 of 19 self-rating resilience measures by Windle, Bennett and Noyes (2011) evaluated the Resilience Scale for Adults (RSA) as one of the best with regard to psychometric ratings. However, it did not receive equally well ratings with regard to cross-cultural validity, which is important as the meaning of resilience may vary across cultures and contexts. Hence, the aim of the present paper was to compare the validity of the RSA across two divergent cultures (Norway and Brazil).

The resilience construct includes multiple levels of protective factors, such as personal resources, impulse control, problem solving abilities, certain qualities in the family, and social or societal support. Protective factors may also sustain normal development or facilitate adaptation better in company with other protective factors rather than separately (Cicchetti & Curtis, 2007; Masten, 2007). Self-report measures of resilience should also capture protective factors at several levels in order to be useful across a wider domain of life circumstances that may compromise mental health.

### The development and validation of the Resilience Scale for Adults (RSA)

The original RSA (Hjemdal, Friborg, Martinussen, & Rosenvinge, 2001) has undergone several stages of development. First, exploratory factor analyses were conducted to identify the central underpinnings of the construct (Friborg et al., 2003). Later, confirmatory factor analytic approaches were used to establish factorial and structural validity (Friborg, Barlaug, Martinussen, Rosenvinge, & Hjemdal, 2005; Friborg, Hjemdal, Martinussen, & Rosenvinge, 2009). The RSA includes 33 items covering six dimensions assessing protective factors at multiple levels: 1) *Perception of self* (Cronbach alpha α = .74), 2) *Planned future* (α = .73), 3) *Social competence* (α = .83), 4) *Structured style* (α = .80), 5) *Family cohesion* (α = .80), and 6) *Social resources* (α = .74) (Hjemdal, Friborg, Stiles, Rosenvinge, & Martinussen, 2006). While the four first factors assess protective factors at a personal level, the two latter assess protective factors at a family and a social level.

The development of the RSA was based on a thorough review of protective factors reported by resilience researchers (Friborg, Hjemdal, Rosenvinge, & Martinussen, 2003; Hjemdal, Friborg, Martinussen, & Rosenvinge, 2001). It discriminates psychiatric patients from healthy controls (Friborg et al., 2003). In the general population, it discriminates between individuals displaying different Bigfive personality profiles (good adjustment vs vulnerability) (Friborg et al., 2005). In an experimental study inflicting pain in the upper arm on the participants, higher RSA scores predicted less levels of pain and stress (Friborg, Hjemdal et al., 2006). In a prospective study, the development of psychiatric symptoms following stressful life events was moderated by the RSA (Hjemdal et al., 2006), hence confirming a buffering effect.

### Studies addressing critiques of the RSA

A critique of the resilience construct has been that it simply represents the counterpart of vulnerability and psychiatric ill-health, thus adding little beyond a proper measurement of negative health. This notion was empirically tested by Friborg et al. (2009) in an analysis of second-order latent factors. The RSA factors related to social competence, family and social resources landed on a different second-order dimension than the variables for psychiatric symptoms, vulnerability and RSA personal competence. The RSA also predicted anxiety and depression symptoms despite controlling for negative stressors and cognitive vulnerability. Taken together, the RSA factors seem to operate both as directly opposing forces to mental health problems as well as independent factors facilitating positive adjustment.

Another challenge with some of the existing resilience measures, as for example the Resilience Scale (Wagnild & Young, 1993) and the Ego-Resiliency Scale (Block and Kremen, 1996) has been their strong relation with personality, thus not predicting adaptive behavior after controlling for personality traits. This critique is also relevant against the RSA. However, in a prediction study of hopelessness, the RSA turned out to be the best predictor even after controlling for the entire NEO-PI-R inventory, as well as several other covariates (Hjemdal, Friborg, & Stiles, 2012). The study indicated that personality traits were indeed important predictors of adaptation, but that the RSA still contributed additionally.

### Cross cultural validations of the RSA

The scale was originally developed in Norway, and has been translated to several other languages. Despite good psychometric properties and adequate construct validity of the RSA, the number of cross-cultural validation studies is low. One of the few studies available by Jowkar et al. (2010) found that the psychometric properties in terms of factorial composition and test score reliability were adequate in an Iranian sample. The RSA also discriminated between an at-risk sample and a matched control, thus supporting the construct validity of a Persian version of the RSA. The second cross-cultural study was conducted in a French speaking Belgium sample (Hjemdal et al. 2011), which basically supported the cross-cultural validity of the RSA. In this particular study only one expected gender difference was found, namely Social Resources. There were no gender differences in Personal Competence in the Iranian sample, which differs from western samples. Studies based on community samples in other countries have shown promising results basically supporting the factor structure in i.e. India (Narayanan, 2007, 2008).

We have received several requests for translation and use of the scale within a Brazilian culture, hence the aim of the present study was to examine whether the RSA would show comparable cross-cultural psychometric properties in a Brazilian context.

### Methodological approaches to cross-cultural validation

Analyses of measurement invariance are well suited for investigating whether people in Brazil ascribe a different meaning to the same set of questions as Norwegians do (Cheung & Renvold, 2009). If deviances between the two cultures exist, invariance analyses may pinpoint exactly at which levels differences are present.

Differences may emerge at a number of different places, but all are not equally relevant for the present study. Form invariance represents the first test and simply asks whether the factor models are comparable across cultures, i.e., are the number of factors and the placement of items on factor scores the same across cultures. The subsequent tests represent further restrictions of this model. Restrictions may be put on factor loadings, intercepts, residual scores, latent means or latent correlation coefficients by requiring them to be equal across the two samples. If the model fit statistics worsen significantly by inflicting such equality constraints, cross-cultural equality may not be implied.

Scalar invariance requires the latent intercepts to be equal. Given equal factor loadings, differences in the intercepts implies that the mean values of the item scores are different, hence indicating that acquiescence as a response bias may be present in one of the cultures (Cheung & Renvold, 2009). However, as individual differences in the latent construct more often are of interest, metric invariance is often a sufficient assumption. It requires the factor loadings to be comparable, and if supported, implies that an equal amount of increase in the raw scores indicate an equal increase on the latent trait, thus individuals from both groups interpret the scale of the item similarly. This would also mean that the beta coefficients from a standard regression analysis would represent the same degree of change in the latent construct and be comparable across both cultures. Invariance in the residuals (latent error) indicates that the measurement reliability of the indicators are comparable, hence indicating that standard errors of the raw mean scores are equal across samples. This test is less relevant as it does not indicate different levels in the measured construct, but that the precision of the items vary. The conventional practice of comparing two summated raw scores using t-tests is based on the undue assumption that all of the above parameters are equal. Finally, and following adjustments for the above differences, it was tested whether the latent mean (the kappa coefficient) of each subscale of the RSA was different between the two countries. If a conventional *t*-test indicates a significant difference for a particular RSA subscale score, whereas the kappa coefficient for the respective subscale score is non-significant, then the raw score difference according to the *t*-test is due to methodological reasons rather than true cross-cultural differences on a construct level.

### Hypothesized findings

Based on previous studies on Norwegian samples (Friborg *et al*., 2005 Hjemdal *et al*., 2006), the six factor structure of the RSA was expected to replicate (representing form invariance). Metric invariance would be supported if the factor loadings are comparable. As it was partly supported in the Norwegian-Belgium cross-cultural validation study, it was expected to be present here as well given the RSA items have a universal property. Invariance in item score reliabilities would be supported if residual variances were comparable. This is seldom the case and also of less concern, and was not expected nor required. Finally, scalar invariance would be supported if the latent intercepts were equal. This is required for cross-cultural comparisons (by making the raw scores directly comparable), but it is not required for a valid use of the scale within a country (by establishing new descriptive norms).

Gender differences have been identified for some factor scores. Males scoring higher on *Perception of self* and women scoring higher on *Social competence* and *Social resources* in Norwegian samples (Friborg & Hjemdal, 2004), and gender differences will be explored in the present study. Werner (1989) also reported that men rated themselves higher on personal competence than women while women generally reported themselves as more skilled in utilizing social support. A meta-analysis exploring gender differences found that men report higher levels of self-esteem and assertiveness, while women generally were more extraversion, trust, gregariousness and nurturance (Feingold, 1994).

Based on previous research (Friborg et al., 2003; Hjemdal et al., 2006) significant positive covariance was expected between the RSA with a Portuguese version of Sense of Coherence (SOC, Antonovsky, 1993), and negative covariance with Hopkins Symptom Checklist (HSCL-25, Derogatis, Lipman, Rickels, Uhlenhuth, & Covi, 1974), thus supporting the construct validity.

## Method

### Participants

A random sample of university students from the “Centro de Filosofia e Ciências Humanas” of the Universidade Federal de Pernambuco in Recife, Pernambuco, Brazil were recruited. In all, 222 participants responded to the questionnaires. One participant was excluded due to more than 90 % missing. Among the remaining 221 participants, 155 were women (*M* = 22.59, *SD* = 4.59) and 62 were men (*M* = 24.77, *SD* = 6.64). Four did not report their gender.

Participants in the Norwegian sample studied at the Norwegian University of Science and Technology, and were recruited from social sciences (73 males and 242 females). Their age ranged from 17 to 44 years (*M* = 22.30, *SD* = 3.24).

### Ethics

The project was performed in accordance with the Declaration of Helsinki, and approved by the Regional Committees for Medical and Health Research Ethics Central Norway and the Research Ethics Committee of the Universidade Federal de Pernambuco. The participants were adults and gave their written informed consent to participation.

### Instruments

Gender and age was collected as demographical information.

*The Hopkins Symptom Check List-25 (HSCL-25)*. The HSCL-25 (Derogatis et al., 1974) is a brief 25-item version of the Symptom Check List (SCL-90-R) (Derogatis, 1983; Derogatis, Lipman, Rickels, Uhlenhuth, & Covi, 1973), that measures depressive and anxiety symptoms on a 4-point Likert scale ranging from 1 (not at all) to 4 (very much). It contains 13 depression items, 10 anxiety items and 2 somatic items and higher scores indicating higher levels of psychiatric/affective symptoms. It has been found to be a valid screening tool for possible clinical depression or anxiety (Glass, Allan, Uhlenhuth, Kimball, & Borinstein, 1978; Hough, Landsverk, & Jacobsen, 1990), also cross-culturally (Hinton, Chen, Tran, Newman, & Lu, 1994; Lavik, Laake, Hauff, & Solberg, 1999; McKelvey & Webb, 1997; Mollica, Wyshak, De Marnefe, Khuon, & Lavelle, 1987; Moum, 1998; Strand et al. 2003).

*Sense of Coherence (SOC-13).* The SOC-13 measures Sense of Coherence (SOC) and is a brief version of the SOC-29 self-report questionnaire (Antonovsky, 1993). Derived from studies of concentration camp survivors from the Second Wold War, it measures a general positive intrapersonal adjustment which is important in preserving good mental health. There are three underlying psychological constructs that comprise Sense of Coherence, namely: comprehensibility (cognitive), manageability (instrumental/behavioural), and meaningfulness (motivational) (see e.g. Eriksson & Lindström, 2005). It uses a seven point semantically differentiated scale with semantic positive and negative at each endpoint. Higher scores indicate higher levels of SOC. The scale has been used in more than 20 countries and been reported to have high reliability (Cronbach’s alphas between .82 and .95) (Antonovsky, 1993; Spadoti, Silva, & Ciol, 2014; Friborg, Hjemdal, Rosenvinge, & Martinussen 2003). Further it has correlated significantly negatively with current depression, experienced stress and trait anxiety (Frenz, Carey, & Jorgensen, 1993; Sammallahti, Holi, Komulainen, & Aalberg, 1996).

*Resilience Scale for Adults (RSA)*. The RSA (Friborg *et al*., 2003; Hjemdal *et al*., 2001) is a 33 item self-report scale for measuring protective resilience factors among adults (Friborg *et al*., 2005; Friborg & Hjemdal, 2004). The reliability and validity of the RSA has been found satisfactory in several studies (e.g., Friborg *et al*., 2005; Friborg & Hjemdal, 2004; Friborg *et al*., 2003; Friborg et al. 2006a; Friborg *et al.,*^2009^; Hjemdal *et al.,*^2006^). It uses a seven point semantic differential scale in which each item has a positive and a negative attribute at each end of the scale continuum (Friborg et al. 2006b). Half of the items are reversely scored in order to reduce acquiescence-biases. Higher scores indicate higher levels of protective resilience factors. Initially, a five-factor structure was reported. Later confirmatory factor analyses indicated a better fit when splitting one of the five factors. The final version has a six factor solution (Friborg *et al*., 2005; Hjemdal *et al.,*^2006^) with factors named: 1) *Perception of self* (Cronbach’s α = .74), 2) *Planned future* (α = .73), 3) *Social competence* (α = .83), 4) *Structured style* (α = .80), 5) *Family cohesion* (α = .80), and 6) *Social resources* (α = .74) (Friborg *et al.,*^2005^; Hjemdal *et al*., 2006). The procedure for translation of the RSA is that two independent persons translate from English to Portuguese, afterwards two new independent persons back-translate from Portuguese to English. The translations are evaluated and the items closest to the original content were chosen. The Portuguese version was piloted to check for comprehensibility.

### Statistics

SPSS 19.0 was used for descriptive statistics, correlations and reliability analyses. As in previous publications on the RSA, all Brazilian item scores were significantly negatively skewed (*Z* ranging from -2.3 to -.9.4), which is a normal phenomenon. Twenty-four of 33 items also indicated a non-normal kurtosis (*Z* ranging from -6.3 to +6.69), and hence, considerable multivariate kurtosis was present (Mardia’s = 61.4, *p* < .001). As non-normal kurtosis biases estimation by narrowing the standard errors of the parameters, an asymptotic covariance matrix was estimated using PRELIS (Jöreskog & Sörbom, 2006) and included as a weight matrix to adjust the error band. Robust maximum likelihood estimation was provided using Satorra-Bentler rescaled chi-square statistics (*SB χ*^*2*^). Following Hu and Bentler (1999) and Marsh et al. (2004), the comparative fit index (CFI) and root mean square residual (RMSEA) were evaluated in addition to *SB χ*^*2*^ when assessing model fit. A CFI > 0.95 and RMSEA < 0.06 indicate a reasonably good model fit. However, as models almost always include some degree of misspecification, which the chi-square statistic easily detects and rejects given a large enough sample, the RMSEA and the CFI index were consulted as well.

Internal consistency was estimated under the assumption of tau-equivalence (Cronbach’s alpha) and non-equivalence (Raykov 2001).

A multigroup CFA approach was taken to determine the degree of invariance in test scores across the Brazilian and the Norwegian sample, as it allows for statistical testing of differences in test parameters across cultures (Byrne, 2010; Ployhart & Oswald 2004). Identification of the models was ensured by fixing the variance of one of the factor loadings to 1, as fixing the factor variances represents a too stringent test of metric equivalence. The factor loading with the smallest non-significant difference between the samples was fixed to 1. Measurement invariance was tested by specifying increasingly restrictive models. First, form invariance was tested by examining whether the same factor model indicated an adequate model fit in both samples (e.g., RMSEA < .06). Second, metric invariance was tested by constraining the factor loadings equal across the samples. Third, invariant measurement errors (equal item score reliability) were examined by constraining the residual variances equal. Fourth, scalar variance was tested by constraining the intercepts (latent mean values) equal. Finally, the kappa parameters were estimated as invariant or free to examine differences in the latent mean values between countries.

As the increasingly restrictive models estimate the same parameters as in the less constrained models, they are nested within the comparison model and have more degrees of freedom. Hence, goodness of fit may be compared statistically by comparing whether the increase in chi-squares is significantly larger than the increase in degrees of freedom. As these tests were based on the rescaled Satorra-Bentler chi-square values, these difference tests were adjusted for non-normality according to instructions by Satorra and Bentler (2001). If significant differences emerged, post-hoc analyses on a factor or an item level were performed to identify the source of misfit. The delta (change) values for the RMSEA and the CFI were also presented. However, the substantive meaning of these is harder to interpret in invariance testing. A simulation study by Chen (2007) indicated ΔRMSEA and ΔCFI values higher than .0139 and -.0030 for loadings, .0124 and -.0038 for intercepts, and .0118 and -.0032 for residuals might be considered as significant. However, as these values were based on eight indicator models (one factor), which is a bit different than the models compared here, we put more weight on the S-B chi-square different tests.

## Results

### Psychometric characteristics of the RSA factor model

As resilience factors covary, all factors were allowed to correlate. The original factor structure validated reasonably well in the Brazilian sample (*SB χ*^*2*^_480_ = 686.5, *p* < .001; *RMSEA* = .044; *CFI* = .960), and even better than in the Norwegian sample (*SB χ*^*2*^_480_ = 902.93, *p* < .001; *RMSEA* = .053; *CFI* = .957). As the model fit was considered acceptable, no further model revision was required.

### Descriptive and correlational statistics

Table 1 presents the means, standard deviations and reliability estimates of the measurement instruments. In the Brasilian sample, Cronbach’s alpha for the total RSA score was .88, and varied between .56 and .79 for the subscale sum scores. The Raykov’s rho reliability estimates were comparable (see Table 1). The statistical associations between the RSA subscales were generally of high magnitude, which was expected and in line with previous reports. Most importantly, comparisons of the Fisher Z transformed correlation coefficients between the RSA subscales across the two cultures did not show any statistical significant differences.

**Table 1:** Means, standard deviations, test score reliability and pearson’s correlations between RSA scores

	Brazil		Norway			Reliability		Correlation coefficients						
	*n* = 222		*n* = 314			Brazil								
	*Mean*	*SD*	*Mean*	*SD*	*g*	α	ρ	1	2	3	4	5	6	7
1 RSA total	5.45	.71	5.32	.71	.18*	.88			.75	.68	.71	.40	.67	.71
2 Perception of self	5.08	1.10	4.90	1.18	.16	.75	.75	75		.52	.46	.19	.34	.35
3 Planned future	5.53	1.14	4.98	1.33	.44***	.67	.66	61	.55		.30	.36	.30	.27
4 Social competence	5.63	.96	5.33	1.05	.30***	.68	.71	68	.45	.39		.09	.33	.55
5 Structured style	4.95	1.19	4.59	1.20	.30***	.56	.57	52	.32	.30	.18		.08	.05
6 Family cohesion	5.29	1.20	5.47	1.06	-.16	.79	.79	66	.28	.14	.21	.21		.56
7 Social resources	6.01	.86	6.16	.76	-.19*	.77	.77	75	.35	.31	.50	.21	.54	

*Note*. *p < .05, ***p < .001, *g* = Hedge’s g (effect size), α = Cronbach’s alpha, ρ = Raykov’s *rho* based on congeneric scores. Correlations coefficients between the RSA subscale scores for the Brazilian sample are presented in the lower diagonal and for Norway in the upper diagonal. Correlations above > .11 are significant at *p* < .05, and above > .16 at *p* < .01

### Mean differences in RSA scores across culture and gender

As we conducted six cross-cultural comparisons, one for each RSA subscale, a *p*-value ≤ .01 was considered necessary. A sum score difference between cultures emerged for the following three subscales (see Table 1 for t-tests and effect size statistics): *Planned future, social competence and structured style*, indicating a higher score on these subscales in Brazil compared with Norway.

In the Brazilian sample, gender differences in one the six RSA subscales emerged, indicating that females reported significantly more social resources than males (*M =* 6.11 vs *M =* 5.79) (*t* = 2.50, *p* = .013).

In the Norwegian sample there were gender differences for three of the six RSA subscales, with men reporting higher scores on *Perception of self* (*M =* 5.25 vs *M =* 4.79) (*t* = -2.95, *p* = .003), and women reporting higher scores on *Social resources* (*M =* 6.26 vs *M =* 5.85) (*t* = 3.63, *p* < .000), and on *Family cohesion* (*M =* 4.94 vs *M =* 4.67) (*t* = 2.56, *p* = .011).

### Confirmatory factor analyses and measurement invariance

Since the original six factor model of the RSA was considered adequate in terms of model fit in both cultures (models M1a and M1b in Table 2), form invariance was adequately supported. The standardized factor loadings from these two models are presented in Table 3. The baseline model, combining the two datasets in a multigroup confirmatory analysis (M2) was also considered adequate in terms of a sufficiently low RMSEA index (.049).

**Table 2:** Evaluations of measurement invariance between Brazil and Norway

Model	Type of test	Compared with	*χ* ^*2*^	*SB χ* ^*2*^	*df*	*ε*_*a*_	*CFI*	Δ*df*	Δ*SB χ* ^*2*^	Δ *ε*_*a*_	*ΔCFI*
M1a	Brazil		937.78	686.47	480	.0441	.9596				
M1b	Norway		1195.42	902.93	480	.0530	.9567				
M2	Baseline (both models)		2133.19	1586.00	960	.0494	.9579				
M3	Factor loadings: λ_all equal_	M2	2183.00	1620.29	987	.0490	.9574	27	34.87	-.0004	-.0004
M4	Item errors: δ_all equal_	M3	2518.85	1747.11	1020	.0516	.9511	33	78.72***	.0026	-.0063
M4a	λ_all equal,_ δ_7 free_	M3	2294.50	1649.03	1013	.0485	.9572	26	36.36	-.0005	-.0002
M5	Intercepts/means: τ_all equal_	M4a	2614.97	1960.31	1046	.0572	.9385	33	320.47***	.0087	-.0187
M5a	λ_all equal,_ δ_7 free,_ τ_22 free_	M4a	2313.82	1671.17	1024	.0486	.9565	11	19.32	.0001	-.0007
M6	Latent means equal: κ_all equal,_ λ_all equal,_ δ_7 free,_ τ_22 free_		2317.73^b^	1675.31	1025	.0487	.9563				
M6a	Latent means different: κ_all free,_ λ_all equal,_ δ_7 free,_ τ_22 free_	M6	2306.69	1660.02	1019	.0484	.9569	6	11.04	-.0003	.0006

*Note*. ^***^ *p* < .001. *SB χ* ^*2*^ = Satorra-Bentler rescaled chi-square, *ε*_*a*_ = Root Mean Square Error of Approximation, CFI = Comparative Fit Index, Δ = change in statistical values. ^b^The model is similar to M5a, except one intercept in the PS factor had to freed up for model identification purposes. λ = factor loadings (lambda), δ = residual error variances (delta), τ = latent intercepts (tau), κ = latent factor means (kappa)

**Table 3:** Standardized Factor Loadings in Both Countries

	Brazil (*n* = 222)							Norway (*n* = 314)					
*Items*	1	2	3	4	5	6		1	2	3	4	5	6
PS 1	.51							.58					
PS 2	.55							.81					
PS 3	.64							.56					
PS 4	.54							.80					
PS 5	.66							.75					
PS 6	.59							.56					
PF 1		.52							.48				
PF 2		.59							.75				
PF 3		.52							.71				
PF 4		.56							.84				
SC 1			.33							.43			
SC 2			.40							.40			
SC 3			.62							.85			
SC 4			.81							.86			
SC 5			.57							.43			
SC 6			.51							.58			
SS 1				.35							.33		
SS 2				.29							.45		
SS 3				.69							.81		
SS 4				.72							.76		
FC 1					.44							.58	
FC 2					.82							.69	
FC 3					.74							.75	
FC 4					.68							.61	
FC 5					.65							.52	
FC 6					.74							.50	
SR 1						.52							.53
SR 2						.87							.63
SR 3						.61							.54
SR 4						.44							.42
SR 5						.82							.73
SR 6						.60							.56
SR 7						.68							.57

*PS* Perception of self, *PF* Positive future, *SC* Social competence, *FC* Family cohesion, *SR* Social resources and *SS* Structured style

The most important test of invariance was the analysis of metric invariance, i.e., equal factor loadings. In model M3 all factor loadings were constrained equal, which did not result in a poorer model fit in terms of the *SB χ*^*2*^ difference test. The ΔRMSEA and ΔCFI were minor.

Equivalence of test score reliability was not supported, as the *SB χ*^*2*^ difference test was significant (model M4 was worse than M3). The ΔCFI also exceeded the desired amount, although the ΔRMSEA was minor. The modification indices were used to identify items showing a significant difference in item score reliabilities between the groups. Seven error variances had to be freed up to achieve invariance between the groups (model 4a). As equivalence in score reliability is a rather stringent test of equivalence and very seldom completely supported in psychological measures, the percentage of items (7 of 33: 21 %) causing non-invariance was considered small.

Scalar invariance is the most stringent test of invariance by demanding all estimated intercepts for the latent scale equal. Support of scalar invariance makes direct comparisons of observed mean score values across countries possible. As expected, it was not supported as evidenced by a significant worsening in the *SB χ*^*2*^ difference test (M5 was worse than M4a). The ΔCFI also confirmed a considerable worsening, although the ΔRMSEA was minor. Non-invariant items were identified by again checking the modification indices. Twenty-two items had to be freed up in order to achieve invariance (M5a not different from M4a). This indicates that observed RSA subscale mean score differences between the countries are confounded mainly by different intercepts and partly by different measurement errors.

Estimation of the kappa coefficients (the latent means) were based on model M5a, which adjust the factor mean scores for differences in intercepts and reliabilities. In the first model (M6) all kappa coefficients were constrained equal, and in the second model (M6a) all were estimated freely. The improvement in model fit was not significant (*SB χ*^*2*^_6_ = 11.04, *p* = .09). Hence, the raw score mean differences between the two cultures as reported in Table 1 are more probably confounded with the different psychometric properties of the scale across cultures rather than reflecting real differences on a construct level.

### Validity of the RSA

As expected the RSA total score correlated significantly negatively with HSCL-25 (*r* = -.38, *p* < .01), and significantly positively with SOC (*r* = .71, *p* < .01). The subscales of the RSA also correlated significantly positively with SOC (ranging from *r* = .25 to .69). Conversely, the RSA subscales (except *Family cohesion*) correlated significantly negatively with HSCL-25 (ranging from *r* = -.16 to -.44).

## Discussion

The evidence of the cross-cultural validity of the RSA is expanding. The main advantage of conducting analyses of factorial invariance is the possibility to examine if the underlying latent constructs remain the same across different samples from different countries. The present results indicated that the six factor structure replicated adequately, thus supporting form invariance. The construct validity coefficients in the Brazilian sample were largely comparable with previously reported findings in Norwegian samples. The factor structure of the RSA has been extensively tested in Norwegian samples, and these studies have consistently supported a six-factor model (e.g. Friborg et al., 2009; Hjemdal et al., 2006). The degree of model misspecification in terms of RMSEA was within acceptable limits, and it was even smaller in the Brazilian sample than in the Norwegian sample (Hjemdal et al., 2006).

The most important test of invariance, namely metric invariance (comparable factor loadings), was supported. This is an important finding because it implies that a one-point higher raw score on the RSA corresponds to an equal amount of change in the latent trait in both Brazil and Norway. Although some of the items showed larger differences in standardized loadings, the overall test were not significant as most differences were negligible. Participants thus interpret the item wording and use the response scale similarly in both countries. The subscale scores from the RSA thus appear to measure variation in the latent traits roughly equivalently between the countries. One may therefore expect that the test scores from the RSA factors in the Brazilian version correlate comparably with other psychological constructs, as has been previously reported in numerous papers on the RSA.

The lack of invariant latent intercepts was of less concern. Nevertheless, it implies that a displacement in scale location between the two countries exists. The intercepts were on average higher in Brazil than in Norway for the factors *Planned future* and *Social competence*, but lower for the external factors *Social resources* and *Family cohesion*. This is partly reflected in Table 1 by indicating comparable differences between the countries on the mean raw score level. The reasons for these differences are not clear, but one possibility is that the numbers on the scale mean different things for individuals in Norway and Brazil without necessarily reflecting real differences on a construct level.

There were gender differences in both samples. In the Norwegian sample such differences were found for the three factors *Perception of self*, *Social resources* and *Family cohesion*, with men scoring higher on the first, and women on the last two. In the Brazilian sample gender differences were only found for *Social resources*, with women scoring higher than men.

The score reliability in the Brazilian sample was however noticeably lower than in Norwegian sample. The Cronbach’s alpha of the total RSA score was .88, but varied between .56 and .79 for the six RSA subscale sumscores. The true scale reliability estimates based on congeneric scores were slightly higher, as expected. In sum, the reliability of three of the factors were satisfactory but rather low for the factors *Planned future*, *Social competence* and *Structured style*. Since the tests for metric invariance also indicated that these factors are measured less strongly, these items should be used with caution in Brazil. The translated version thus captures less variance on the construct level than the original version. This may be circumvented by using larger samples. Nevertheless, the Brazilian version appears less suited for individual assessment. Future studies are needed to explore if the internal consistency remains low, or that the finding may be related to the sample.

The construct validity of the RSA was supported in the Brazilian sample as the correlations were as expected. The total score significantly negative correlation with HSCL-25, and significant positive correlation with SOC. Correlations were within moderate to strong in size. These results are in accordance with the construct of resilience as representing the presence of protective recourses associated with good adaptation and mental health as well as the relative absence of psychiatric symptoms found in Norwegian samples (Friborg et al., 2003, 2009; Hjemdal et al., 2006). The RSA-factors all showed significant positive correlations with SOC, which were between strong and medium. All showed significant negatively correlations with HSCL-25, in the range between medium to small, with the exception of *Family cohesion* which was non-significant.

The major limitations of the present study is the young age of the participants and that all were university students and unbalanced proportion of women and men in the sample, which implies caution with regard to generalizing the results to the general adult population in Brazil. Further validity studies of the RSA on more heterogeneous samples in terms of age and occupation may address this uncertainty.

## Conclusions

The six-factor structure of the RSA was confirmed using confirmatory factor analysis, and the RSA scores had a pattern of intercorrelations similar to that of those reported previously. All together, the results support the psychometric properties and the validity of the RSA in a Brazilian sample.

The results also indicate that the protective factors included in the RSA may be relevant across cultures. Further studies are needed to explore if these protective resilience factors are universals shared by other cultures.
